# Risk Factors Affecting Alternate Segregation in Blastocysts From Preimplantation Genetic Testing Cycles of Autosomal Reciprocal Translocations

**DOI:** 10.3389/fgene.2022.880208

**Published:** 2022-06-02

**Authors:** Pingyuan Xie, Liang Hu, Yangqin Peng, Yue-qiu Tan, Keli Luo, Fei Gong, Guangxiu Lu, Ge Lin

**Affiliations:** ^1^ Hunan Normal University School of Medicine, Changsha, China; ^2^ National Engineering and Research Center of Human Stem Cells, Changsha, China; ^3^ Hunan International Scientific and Technological Cooperation Base of Development and Carcinogenesis, Changsha, China; ^4^ NHC Key Laboratory of Human Stem Cell and Reproductive Engineering, Institute of Reproductive and Stem Cell Engineering, School of Basic Medical Science, Central South University, Changsha, China; ^5^ Clinical Research Center for Reproduction and Genetics in Hunan Province, Reproductive and Genetic Hospital of CITIC-Xiangya, Changsha, China

**Keywords:** reciprocal translocation, PGT-SR, meiotic segregation patterns, next-generation sequencing, blastocysts

## Abstract

Reciprocal translocations are the most common structural chromosome rearrangements and may be associated with reproductive problems. Therefore, the objective of this study was to analyze factors that can influence meiotic segregation patterns in blastocysts for reciprocal translocation carriers. Segregation patterns of quadrivalents in 10,846 blastocysts from 2,871 preimplantation genetic testing cycles of reciprocal translocation carriers were analyzed. The percentage of normal/balanced blastocysts was 34.3%, and 2:2 segregation was observed in 90.0% of the blastocysts. Increased TAR1 (ratio of translocated segment 1 over the chromosome arm) emerged as an independent protective factor associated with an increase in alternate segregation (p = 0.004). Female sex and involvement of an acrocentric chromosome (Acr-ch) were independent risk factors that reduced alternate segregation proportions (p < 0.001). Notably, a higher TAR1 reduced the proportion of adjacent-1 segregation (p < 0.001); a longer translocated segment and female sex increased the risk of adjacent-2 segregation (p = 0.009 and p < 0.001, respectively). Female sex and involvement of an Acr-ch enhanced the ratio of 3:1 segregation (p < 0.001 and p = 0.012, respectively). In conclusion, autosomal reciprocal translocation carriers have reduced proportions of alternate segregation in blastocysts upon the involvement of an Acr-ch, female sex, and lower TAR1. These results may facilitate more appropriate genetic counseling for couples with autosomal reciprocal translocation regarding their chances of producing normal/balanced blastocysts.

## Introduction

Reciprocal translocations are the most common structural chromosomal reorganizations in humans and typically involve two chromosomes, each with one breakpoint to generate two distal segments that are interchanged (Morin et al., 2017). Balanced translocation carriers are commonly phenotypically normal, and >6% of reciprocal carriers have a variety of symptoms, such as autism, intellectual disabilities, or congenital abnormalities (Warburton, 1991). Reciprocal translocations are associated with reproductive problems, such as subfertility, infertility, and recurrent miscarriage, because of genomic imbalance in the gametes (Scriven et al., 1998; Morin et al., 2017; Fatemi et al., 2021). The prevalence of reciprocal translocation is approximately 0.14% in newborns (Nielsen and Wohlert, 1991), 1.2% in infertile men with azoospermia (Stern et al., 1999; Clementini et al., 2005), and 2.4–6.9% in couples with recurrent miscarriage (Clifford et al., 1994; Stern et al., 1999).

During meiosis, the two translocated chromosomes and their two homologous normal chromosomes form a quadrivalent (Scriven et al., 1998; Tease et al., 2002). In anaphase I, the quadrivalent structure is segregated to the daughter cells through one of the following five modes: 1) alternate segregation, including segregation of a normal chromosome complement and balanced rearrangement into different poles; 2) adjacent-1 segregation, in which homologous centromeres pass to the opposite poles and lead to monosomy for one translocated segment and trisomy for the other; 3) adjacent-2 segregation, in which homologous centromeres are segregated together resulting in trisomy for one centric segment and monosomy for the other; 4) 3:1 segregation, wherein three out of four chromosomes are segregated together and may result in trisomy or monosomy; and 5) 4:0 segregation, wherein all four chromosomes are separated into one pole, leading to double-trisomy or double-monosomy. Owing to recombination, 32 possible gametes with different karyotypes may occur, and only two of these, which arise from the alternate segregation mode, can have normal/balanced genetic content (Scriven et al., 1998).

Preimplantation genetic testing (PGT) has been a common technique to exclude unbalanced embryos for transfer since 1998 (Munne et al., 1998; (Pierce et al., 1998). Originally, fluorescence *in situ* hybridization (FISH) was used to analyze the segregation mode in the spermatozoon or biopsied blastomeres (Anton et al., 2008; Lim et al., 2008). More recently, with the development of whole-genome amplification (WGA) and comprehensive chromosome screening (CCS), including single nucleotide polymorphism (SNP) arrays, comparative genomic hybridization (CGH) arrays, and next-generation sequencing (NGS) (Tan et al., 2014; Idowu et al., 2015), all components of chromosomes can be analyzed simultaneously.

Extensive studies on meiotic segregation patterns of sperm, blastomeres, and blastocysts have identified several factors that may influence the unbalanced rearrangement of reciprocal translocations, including carrier’s sex and age, location of the breakpoints, chromosome type, and quadrivalent structure (Munne, 2005; Anton et al., 2008; Zhang et al., 2018; Zhang et al., 2019a; Zhang et al., 2019b). Gamete results indicate a very wide range of chromosomal abnormalities, depending on the translocation, ranging from 18 to 82% abnormal sperm (Munne, 2005). A previous study suggested that a sperm FISH analysis prior to PGD is not a reliable predictor of the PGT outcome (Haapaniemi Kouru et al., 2017). Recently, several studies have assessed meiotic segregation patterns in blastocysts using CCS, even though controversial results could be achieved (Zhang et al., 2018; Zhang et al., 2019b; Wang et al., 2019). Wang et al. (2019) demonstrated that segregation modes in blastocysts could be affected by the prsence of acrocentric chromosomes and terminal breakpoints, rather than by the carrier’s sex. Zhang et al. (2019b) suggested that an acrocentric chromosome (Acr-ch) involved in translocation interacts with the carrier’s sex to influence the proportion of alternate segregation for normal or balanced chromosome contents during meiotic segregation in autosomal reciprocal translocations.

In the present study, our aim was to evaluate the factors that affect alternate segregation and to provide more appropriate genetic counseling for couples with autosomal reciprocal translocation regarding their chances of producing normal/balanced blastocysts. The impact factors were analyzed according to the carrier’s sex, age, translocated chromosome type, and the location of translocated chromosome breakpoints using NGS.

## Materials and Methods

### Study Subjects

The study was approved by the Ethics Committee of the Reproductive and Genetic Hospital of CITIC-Xiangya.

We enrolled 2,253 couples with autosomal reciprocal translocations, whose blastocysts were tested using NGS, from October 2013 to December 2019 from the Reproductive and Genetic Hospital of CITIC-Xiangya. G-banding chromosome analysis of cultured lymphocytes from peripheral blood was performed at our hospital or other certified organizations. The karyotypes in this report are described according to the ISCN 2013 guidelines. All individuals had a history of infertility, recurrent spontaneous abortion, or pregnancy with chromosomal anomalies.

### PGT-SR Procedure

Preimplantation genetic testing for chromosomal structural rearrangement (PGT-SR) was performed at the Reproductive & Genetic Hospital of CITIC-Xiangya, as previously described. In brief, ovarian stimulation was conducted based on the patient’s status. Fertilization was assessed at 16–18 h post-intracytoplasmic sperm injection (ICSI) by visualization of two pronuclei and two polar bodies. All embryos were cultured in sequential media (G1 and G2; Vitrolife) to the blastocyst stage under 6% CO_2_, 5% O_2_, and 89% N_2_ in a Cook mini-incubator. Trophectoderm (TE) biopsy was performed on day 5 or day 6, and approximately five cells were collected after zona pellucida dissection and hatching using a laser (ZILOS-tk, Hamilton Thorne). The blastocysts were then cryopreserved as previously described (Zhang et al., 2016).

Two NGS platforms were used in this study. For the initial 2,345 trophectoderm cells (from October 2013 to May 2015), whole-genome amplification (WGA) was performed using the WGA4 GenomePlex Single Cell Whole Genome Amplification kit (Sigma-Aldrich, St. Louis, MO, United States). The Illumina HiSeq2000 platform was used for NGS, and approximately 5–10 million single-end (SE) 50 bp reads were obtained for each sample. For the remaining 8,501 trophectoderm cells, WGA was performed using the REPLI-g Single Cell Kit (Qiagen, Hilden, Germany). The Illumina NextSeq platform was used for NGS, and a 10 million SE 75 bp reads were obtained for each sample. Copy number variations larger than 1–4 megabase (>1 Mb) could be detected (Tan et al., 2014; Zhou et al., 2018). The NGS-PGT platform results were confirmed using FISH or SNP arrays before clinical application, as described in our previous studies (Tan et al., 2013; Zhang et al., 2013).

### Embryo Transfer

One or two cryopreserved embryos could be transferred, and only euploid blastocysts were selected for transfer. No blastocyst transfer was performed with an endometrium of < 7 mm thickness.

### Statistical Analysis

The distribution of patient demographics and cytogenetic characteristics was analyzed using the Kolmogorov–Smirnov test. Continuous variables were expressed as mean ± standard deviation (SD). Categorical variables were described as the frequency and percentage. One-way analysis of variance (ANOVA) or Wilcoxon rank-sum test (alternate segregation group versus non-alternate segregation group) or Kruskal–Wallis H test (alternate segregation group versus adjacent-1 segregation group versus adjacent-2 segregation group versus 3:1 segregation group versus ND group) was used for comparison according to the distribution of continuous variables; chi-square or Fisher’s exact tests were used to compare differences between categorical variables.

To evaluate associations of the patient demographics and cytogenetic characteristics with the embryo separation patterns, we utilized a multivariate generalized linear mixed model (GLMM) with random intercepts to account for multiple embryos from the same woman. A binomial distribution with a logit link function (alternate (balanced) segregation group versus unbalanced segregation group) or a multinomial distribution with a logit link function (alternate segregation group versus adjacent-1 segregation group versus adjacent-2 segregation group versus 3:1 segregation group versus not determined (ND) group) was specified for the embryo segregation mode. Covariates with statistically significant differences in univariate GLMM were considered for inclusion in the multivariate GLMM. The following covariates were considered for inclusion in the univariate GLMM: carrier sex, age, and chromosomes involved in the translocation (chromosomes #1 and #2 corresponding to the small and large chromosomes, respectively, according to the guidelines of the International System for Human Cytogenetic Nomenclature (ISCN) 2016). In addition, the inclusion of an Acr-ch, size of the involved chromosomes, size of the translocated segments (TS1 and TS2), size of the centric segments (CS1 and CS2), ratio of the longest and shortest translocated segments (TSR), ratio of the longest and shortest centric segments (CSR), and ratio of the translocated segment over the chromosome arm (TAR) were analyzed. The location of breakpoints was identified by the PGT-SR results of the karyotype; the length of both centric and translocated segments (CS and TS) was measured according to the copy number variations of the unbalanced embryos. TSR, CSR, and TAR were then calculated separately.

A receiver operating characteristic (ROC) curve was used to discriminate the predictive values of the patient demographics and clinical variables in a multivariate GLMM for the embryo separation mode (balanced group versus unbalanced group).

The Cochran–Mantel–Haenszel (CMH) test was utilized to explore the interaction effect of carrier sex and the involvement of an Acr-ch on the likelihood of every single embryo.

All statistical analyses were performed using SPSS 25.0 software (IBM, Armonk, NY, United States), and a two-sided p-value < 0.05 was considered statistically significant.

## Results

### Patient Demographics and Clinical Characteristics

This study included 1,028 male and 1,225 female autosomal reciprocal translocation carriers. There were no significant differences in demographic characteristics between female and male carriers. In total, 31,295 oocytes originating from 2,871 oocyte retrieval cycles underwent ICSI, and 12,328 (39.4%) embryos were developed to blastocysts on days 5 or 6. Among 12,328 TE-biopsied embryos, 10,876 (88.2%) were tested and 10,846 were diagnosed successfully (Table 1); the percentage of normal/balanced blastocysts was 34.3% (3,716/10,846). The clinical pregnancy rate and delivery rate in the male carrier group were significantly higher than those in the female carrier group (p < 0.05) (Table 1).

**TABLE 1:** Clinical characteristics and preimplantation genetic testing for chromosomal structural rearrangement outcomes of reciprocal translocation heterozygotes.

Parameter	Female carrier	Male carrier	p-value	Total
Patient number	1,028	1,225		2,253
Cycles	1,583	1,288		2,871
Female age (years)	29.97 ± 4	30.16 ± 4.04	0.488	29.87 ± 3.78
Male age (years)	31.93 ± 4.8	32.1 ± 5.04	0.151	31.81 ± 4.79
Retrieved oocytes	21,157 (13.4)	17,526 (13.6)	NS	38,684
Injected oocytes	17,175 (10.9)	14,119 (11.0)	NS	31,295
ICSI cleavage embryos	15,175 (9.6)	12,447 (9.7)	NS	27,622
Biopsied embryos	6,743 (4.3)	5,585 (4.3)	NS	12,328
Diagnosed embryos	5,995 (3.8)	4,851 (3.8)	NS	10,846
Transferable normal embryos	1918	1798		3,716
Abnormal embryos	4,077	3,053		7,130
Transferred embryos	876	852		1728
Embryo transfer cycles	830	730		1,560
Clinical pregnancies (%)	514 (61.9%)	526 (72.1%)	<0.001	1,040 (66.7%)
Spontaneous abortions (%)	76 (9.2%)	87 (11.9%)	0.082	175 (11.2%)
Deliveries (%)	437 (52.7%)	438 (60%)	0.004	875 (56.1%)
Ectopic pregnancies	1	1		

All data are n values unless stated otherwise. The results in parentheses are the mean oocyte or embryo numbers in each retrieved cycle.

### Segregation Pattern Analysis

The segregation patterns were determined using PGT-SR results from translocation carrier individuals. Overall, among 10,864 diagnosed blastocysts, 2:2 segregation was observed in 9,751 (90.0%) blastocysts, of which alternate segregation was found as the most frequent segregation pattern (46.1%, 5,005/10,846), followed by adjacent-1 segregation (31.3%, 3,397/10,846) and adjacent-2 segregation (12.4%, 1,349/10,846). The details (karyotype and segregation pattern) are shown in Supplementary Table S1. The frequencies of 3:1 segregation and 4:0/other segregation patterns were 5.4% (585/10,846) and 4.7% (510/10,864), respectively. Among all 4:0/other segregation products, only four 4:0 products were identified, and most were classified as undetermined segregation products; therefore, we concentrated on the 2:2 and 3:1 segregation pattern.

### Acr-ch Involved in the Translocation, Carrier Sex, and Lower TAR1 Could Reduce the Proportion of Balanced Embryos

As only alternate segregation could produce normal or balanced gametes, we first analyzed the factors that could affect the proportion of alternate segregation. The blastocysts were divided into balanced (alternate segregation) and unbalanced (non-alternate segregation) groups according to their segregation patterns. The results of the univariate and multivariate GLMMs are presented in Table 2. All variables with p < 0.05 were used for multivariate analysis, including the length of the involved chromosomes, TAR1, CS2, carrier sex, and involvement of an Acr-ch. The multivariate analysis results indicated that TAR, carrier sex, and involvement of an Acr-ch could significantly influence the proportion of alternate segregation. In particular, 1) increased TAR1 reduced the non-alternate segregation ratio (odds ratio (OR), 0.806 [95% confidence interval (CI), 0.696, 0.933]; p = 0.004), female sex (OR, 1.293 [95% CI, 1.187, 1.408]; p < 0.001), and involvement of an Acr-ch (OR, 1.208 [95% CI, 1.089, 1.339]; p < 0.001) enhanced the proportion of non-alternate segregation. Furthermore, a ROC curve was used to evaluate the predictive values of alternate segregation. The values of area under the curve (AUC), sensitivity, and specificity were 0.708, 84.4%, and 40.3%, respectively (Supplementary Figure S1).

**TABLE 2:** Univariable and multivariable generalized linear mixed model analysis for unbalanced translocation.

	Univariate generalized linear mixed model (GLMM)		Multivariable generalized linear mixed model (GLMM)	
	OR (95% CI)	p	OR (95% CI)	p
Size 1	0.999 (0.998,1.000)	0.006		
TAR1	0.823 (0.711,0.954)	0.01	0.806 (0.696,0.933)	0.004
CS2	0.999 (0.998,1.000)	0.029		
Sex				
Female	1.295 (1.189,1.411)	<0.001	1.293 (1.187,1.408)	<0.001
Male	Reference		Reference	
Acr-ch				
With	1.22 (1.115, 1.333)	<0.001	1.208 (1.089,1.339)	<0.001
Without	Reference		Reference	

Acr-ch, acrocentric chromosome;

Size 1, size of the longer chromosome involved;

CS 2, size of the centric segments of the shorter chromosome involved;

TAR1, the ratio of the translocated segment over the chromosome arm of the longer chromosome.

**FIGURE 1:**
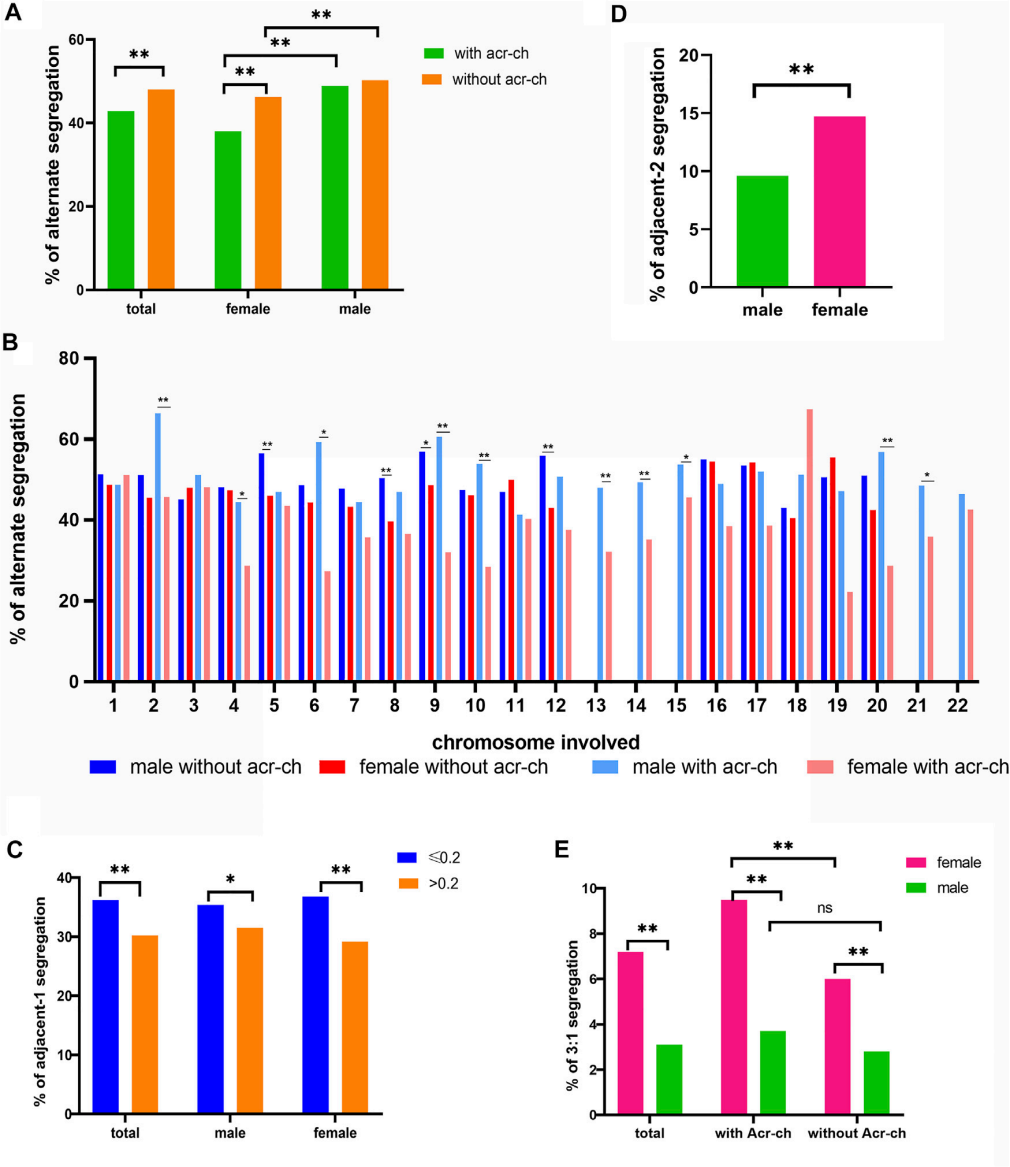
Factors influencing meiotic segregation patterns in blastocysts for reciprocal translocation carriers. **(A)** Alternate segregation proportions in male and female carriers according to the type of chromosome involved (Acr-ch and acrocentric chromosome). **(B)** Alternate segregation proportions in male and female carriers according to the chromosome involved (Acr-ch and acrocentric chromosome). **(C)** Proportion of adjacent-1 segregation under different values of TAR1 and the carrier’s sex group (TAR1, the ratio of translocated segment 1 over the chromosome arm). **(D)** Proportion of adjacent-2 segregation in female and male carriers with reciprocal translocation. **(E)** Proportion of 3:1 segregation with different sex and chromosome types involved (Acr-ch and acrocentric chromosome).

The CMH test was employed to further assess the effects of a carrier’s sex and involvement of an Acr-ch on the likelihood of each single embryo in an unbalanced rearrangement. The results were summarized as follows (Table 3 and Figure 1A): 1) the proportion of blastocysts carrying an unbalanced rearrangement was higher in the female group than that in the male group, both in carriers with an Acr-ch [62% versus 51.1%, p < 0.001, OR = 1.561, 95% CI (1.374, 1.775)] and without an Acr-ch [53.8% versus 49.8%, p = 0.001, OR = 1.176, 95% CI (1.07, 1.293)]. 2) The risk of an embryo with an unbalanced rearrangement was higher in carriers with an Acr-ch than in those without an Acr-ch in the female group [62e % versus 53.8%, p < 0.001, OR = 1.403, 95% CI (1.26, 1.563)], rather than in the male group (51.1% versus 49.8%, p = 0.353). The data were further stratified according to the chromosomes involved; in the Acr-ch group, the proportion of alternate segregation in female carriers, involving chr-2, -4, -6, -9, -10, and -20, was significantly lower than that in the corresponding male carriers (p < 0.05). However, in the non-Acr-ch group, the proportion of alternate segregation in female carriers, involving chr-5, -8, -9, and -12 (p < 0.05), was significantly lower than that in the corresponding male carriers (Figure 1B and Supplementary Table S2).

**TABLE 3:** Analysis of balanced and unbalanced segregation patterns according to carrier sex and chromosome type.

		Unbalanced	Balanced	p	OR (95%CI)	p*
With Acr-ch	Female	1,334 (62%)	816 (38%)	<0.001	1.561 (1.374,1.775)	<0.001
	Male	891 (51.1%)	851 (48.9%)			
Without Acr-ch	Female	2069 (53.8%)	1776 (46.2%)	0.001	1.176 (1.07,1.293)	
	Male	1,547 (49.8%)	1,562 (50.2%)			
Female	With Acr-ch	1,334 (62%)	816 (38%)	<0.001	1.403 (1.26,1.563)	<0.001
	Without Acr-ch	2069 (53.8%)	1776 (46.2%)			
Male	With Acr-ch	891 (51.1%)	851 (48.9%)	0.353	1.057 (0.94,1.189)	
	Without Acr-ch	1,547 (49.8%)	1,562 (50.2%)			

* p for tests of homogeneity of the odds ratio.

Acr-ch, acrocentric chromosome.

### Factors Influencing the Adjacent-1, Adjacent-2, and 3:1 Segregation Patterns

We identified several factors that could influence the proportions of adjacent-1, adjacent-2, and 3:1 segregation patterns (Table 4). In particular, the proportion of adjacent-1 segregation was affected by the TAR1, and a greater TAR1 reduced the proportion of adjacent-1 segregation [OR = 0.316, 95% CI (0.174, 0.575), p < 0.001]. For making simplification, we divided the TAR1 into TAR1 ≤ 0.2 group and TAR1 > 0.2 group. The results indicated that the proportion of adjacent-1 segregation was significantly higher in TAR1 ≤ 0.2 group than in TAR1 > 0.2 group (36.2% versus 30.2%, p < 0.001) and both in female subgroup (36.8% versus 29.2%, p < 0.001) and male subgroup (35.4% versus 31.5%, p < 0.05) (Figure 1C and Supplementary Table S3). The proportion of adjacent-2 segregation was influenced by the TS1 and carrier’s sex, and longer TS1 and female sex increased the proportion of adjacent-2 segregation [OR = 1.014, 95% CI(1.003, 1.024), p = 0.009 and OR = 1.66, 95% CI(1.427, 1.931), p < 0.001, respectively] (Figure 1D and Supplementary Table S4); besides, the proportion of 3:1 segregation was affected by a carrier’s sex and involvement of an Acr-ch, wherein female sex and involvement of an Acr-ch increased the proportion of 3:1 segregation [OR = 2.643, 95% CI (2.147, 3.253), p < 0.001 and OR = 1.387, 95% CI (1.075, 1.789), p = 0.012, respectively] (Table 4; Figure 1E and Supplementary Table S5).

**TABLE 4:** Multivariable generalized linear mixed model analysis.

	Multivariable generalized linear mixed model (GLMM)	p
	OR (95% CI)	
Adjacent-1 vs. alternate		
TAR1	0.316 (0.174,0.575)	<0.001
Adjacent-2 vs. alternate		
TS1	1.014 (1.003,1.024)	0.009
Carrier’s sex		
Female	1.66 (1.427,1.931)	<0.001
Male	Reference	
3:1 vs. alternate		
Carrier’s sex		
Female	2.643 (2.147,3.253)	<0.001
Male	Reference	
Acrocentric chromosome		
With	1.387 (1.075,1.789)	0.012
Without	Reference	

## Discussion

After GLMM analysis of 10,846 biopsied blastocysts from 2,253 autosomal reciprocal translocation individuals, we found that carrier’s sex, involvement of an Acr-ch, and lower TAR1 were independent risk factors that may be associated with the proportion of alternate segregation in autosomal reciprocal translocation. Our results also indicated that the most common pattern was alternate segregation (46.1%) in blastocysts, followed by adjacent-1 segregation. The percentage of normal/balanced embryos in diagnosed blastocysts in the present study was 34.3%, which was similar to that reported in several recently conducted studies (Idowu et al., 2015; Zhang et al., 2018; Zhang et al., 2019b; Wang et al., 2019).

Numerous studies have analyzed the meiotic segregation patterns in gametes or embryos from reciprocal translocation carriers and have demonstrated that the carrier’s sex and age and the location of breakpoints might influence the meiotic segregation patterns, though their results were somewhat controversial (Benet et al., 2005; Lim et al., 2008; Lledo et al., 2010; Ye et al., 2012; Zhang et al., 2018). Regarding the carrier’s sex and involvement of an Acr-ch, studies concentrating on both gametes and embryos suggested that a carrier’s sex influenced the meiotic segregation patterns, and that male carriers have a significantly higher proportion of alternate segregation (Munne et al., 1998; Lin et al., 2021). However, several research studies reported that the proportions of adjacent-1, adjacent-2, or 3:1 segregation patterns were markedly different in male and female carriers, whereas this outcome was not observed in alternate segregation (Ko et al., 2010; Lledo et al., 2010; Ye et al., 2012; Zhang et al., 2018). Previous studies have indicated that participation of an Acr-ch in reorganization or a very asymmetrical configuration of the quadrivalent favored 3:1 segregation owing to the extremely small length of their arms, and thus marked asymmetry of the quadrivalent (Benet et al., 2005; Lim et al., 2008). Furthermore, the percentage of adjacent-1 products from a quadrivalent with an Acr-ch has been demonstrated to be significantly higher than that without an Acr-ch, and no significant differences in other segregation patterns have been identified (Zhang et al., 2018). The translocations with an Acr-ch exhibited a notably higher frequency of 3:1 segregation, whereas there was no significant difference in alternate segregation (Wang et al., 2019).

In contrast to a previous study, we observed no influence of a carrier’s age on the meiotic segregation patterns. Overall, we found that a carrier’s sex, involvement of an Acr-ch, and location of breakpoints may influence the proportion of segregation patterns. Furthermore, the proportion of alternate segregation was significantly higher in male carriers than that in female carriers, particularly when an Acr-ch could interact with chr-2, -4, -6, -9, -10, and -20. Moreover, the proportion of alternate segregation was remarkably higher in female carriers with an Acr-ch than that in female carriers without an Acr-ch, rather than that in male carriers. Furthermore, the proportion of adjacent-2 and the 3:1 segregation patterns was significantly higher in female carriers than that in male carriers, although this finding was not confirmed for adjacent-1 segregation. In addition, the proportion of 3:1 segregation was notably higher in carriers with an Acr-ch than in those without an Ach-ch. Finally, our findings suggest that TAR1 could influence segregation patterns, wherein lower TAR1 could increase the proportion of adjacent-1 segregation and decrease the proportion of alternate segregation. Refer to why only TAR1 but not TAR2 could influence the segregation pattern needed further study.

Compared with previous studies, the present study possesses several strengths. First, the PGT-SR results of 10,846 blastocysts enabled statistical analysis of a relatively large number of samples in each subgroup. Second, analysis of GLMMs was advantageous because these models provided multiple observations per patient, while accounting for within-person correlations in the outcomes. These models could provide unbiased estimates in the presence of an unbalanced design (different numbers of embryos per cycle were contributed by each female patient) when imbalance in the number of embryos was not fully randomized, and the lack of balance could be accurately predicted by all measured covariates in an adjusted model. This study also has several limitations. First, our segregation pattern analysis was based on blastocysts, which may not reflect the actual segregation pattern in gametes. Furthermore, as only four embryos were diagnosed initially, approximately 1,400 blastocysts were not diagnosed in 2,871 cycles, which might have caused bias in the results.

A remarkably higher proportion of alternate segregation for normal or balanced chromosome contents was also observed in the blastocysts of male carriers than that in those of female carriers among Robertsonian translocations and pericentric inversions (Zhang et al., 2019a; Xie et al., 2019), suggesting that the checkpoints in oogenesis are not as stringent as those in spermatogenesis. The different spatial characteristics of the quadrivalent (i.e., involvement of an Acr-ch) could affect synaptic recombination in human oocytes and spermatocytes. However, understanding the detailed underlying mechanism requires further investigations.

NGS-PGT is widely used in PGT-SR to exclude unbalanced translocations and *de novo* chromosomal aberrations. However, in routine PGT, balanced and normal embryos cannot be distinguished by CCS, and recently developed techniques may allow distinction of inversion carrier embryos from those with normal chromosomes (Hu et al., 2016; Wang et al., 2017; Xu et al., 2017).

In conclusion, carrier’s sex, involvement of an Acr-ch, and location of breakpoints may influence the segregation patterns in blastocysts. In addition, involvement of an Acr-ch, female sex, and lower TAR1 as independent risk factors can reduce the proportion of alternate segregation. These results may provide more appropriate information for couples with autosomal reciprocal translocation.

## Data Availability

The original contributions presented in the study are included in the article/Supplementary Material, further inquiries can be directed to the corresponding author.

## Supplementary Material

Supplementary Figure S1 Receiver operating characteristic (ROC) curve was used to evaluate the predictive values of alternate segregation patterns.

## References

[B1] Anton E. Vidal F. Blanco J. (2008). Reciprocal Translocations: Tracing Their Meiotic Behavior. Genet. Med. 10, 730–738. 10.1097/gim.0b013e318187760f 18813133

[B2] Benet J. Oliver-Bonet M. Cifuentes P. Templado C. Navarro J. (2005). Segregation of Chromosomes in Sperm of Reciprocal Translocation Carriers: a Review. Cytogenet. Genome Res. 111, 281–290. 10.1159/000086901 16192706

[B3] Clementini E. Palka C. Iezzi I. Stuppia L. Guanciali-Franchi P. Tiboni G. M. (2005). Prevalence of Chromosomal Abnormalities in 2078 Infertile Couples Referred for Assisted Reproductive Techniques. Hum. Reprod. 20, 437–442. 10.1093/humrep/deh626 15567875

[B4] Clifford K. Rai R. Watson H. Regan L. (1994). Pregnancy: An Informative Protocol for the Investigation of Recurrent Miscarriage: Preliminary Experience of 500 Consecutive Cases. Hum. Reprod. 9, 1328–1332. 10.1093/oxfordjournals.humrep.a138703 7962442

[B5] Fatemi N. Varkiani M. Ramezanali F. Babaabasi B. Ghaheri A. Biglari A. (2021). Risk Factors Associated with Recurrent Pregnancy Loss and Outcome of Pre-implantation Genetic Screening of Affected Couples. Int. J. Fertil. Steril 15, 269–274. 10.22074/IJFS.2021.137626.1027 34913295 PMC8530214

[B6] Haapaniemi Kouru K. Malmgren H. White I. Rodriguez Sanchez A. Syk Lundberg E. (2017). Meiotic Segregation Analyses of Reciprocal Translocations in Spermatozoa and Embryos: No Support for Predictive Value Regarding PGD Outcome. Reprod. BioMedicine Online 34, 645–652. 10.1016/j.rbmo.2017.02.013 28336162

[B7] Hu L. Cheng D. Gong F. Lu C. Tan Y. Luo K. (2016). Reciprocal Translocation Carrier Diagnosis in Preimplantation Human Embryos. EBioMedicine 14, 139–147. 10.1016/j.ebiom.2016.11.007 27840008 PMC5161423

[B8] Idowu D. Merrion K. Wemmer N. Mash J. G. Pettersen B. Kijacic D. (2015). Pregnancy Outcomes Following 24-chromosome Preimplantation Genetic Diagnosis in Couples with Balanced Reciprocal or Robertsonian Translocations. Fertil. Sterility 103, 1037–1042. 10.1016/j.fertnstert.2014.12.118 25712573

[B9] Ko D. S. Cho J. W. Park S. Y. Kim J. Y. Koong M. K. Song I. O. (2010). Clinical Outcomes of Preimplantation Genetic Diagnosis (PGD) and Analysis of Meiotic Segregation Modes in Reciprocal Translocation Carriers. Am. J. Med. Genet. A. 152A, 1428–1433. 10.1002/ajmg.a.33368 20503317

[B10] Lim C. K. Cho J. W. Song I. O. Kang I. S. Yoon Y.-D. Jun J. H. (2008). Estimation of Chromosomal Imbalances in Preimplantation Embryos from Preimplantation Genetic Diagnosis Cycles of Reciprocal Translocations with or without Acrocentric Chromosomes. Fertil. Sterility 90, 2144–2151. 10.1016/j.fertnstert.2007.10.035 18440525

[B11] Lin L. Chen X. Wang J. Li R. Ding C. Cai B. (2021). Effect of Carriers' Sex on Meiotic Segregation Patterns and Chromosome Stability of Reciprocal Translocations. Reprod. BioMedicine Online 43, 1011–1018. 10.1016/j.rbmo.2021.08.017 34654612

[B12] Lledo B. Ortiz J. A. Morales R. Ten J. De La Fuente P. E. Garcia-Ochoa C. (2010). The Paternal Effect of Chromosome Translocation Carriers Observed from Meiotic Segregation in Embryos. Hum. Reprod. 25, 1843–1848. 10.1093/humrep/deq111 20511301

[B13] Morin S. J. Eccles J. Iturriaga A. Zimmerman R. S. (2017). Translocations, Inversions and Other Chromosome Rearrangements. Fertil. Sterility 107, 19–26. 10.1016/j.fertnstert.2016.10.013 27793378

[B14] Munné S. (2005). Analysis of Chromosome Segregation during Preimplantation Genetic Diagnosis in Both Male and Female Translocation Heterozygotes. Cytogenet. Genome Res. 111, 305–309. 10.1159/000086904 16192709

[B15] Munné S. Morrison L. Fung J. Márquez C. Weier U. Bahçe M. (1998). Spontaneous Abortions Are Reduced after Preconception Diagnosis of Translocations. J. Assist. Reprod. Genet. 15, 290–296. 10.1023/a:1022544511198 9604762 PMC3454766

[B16] Nielsen J. Wohlert M. (1991). Chromosome Abnormalities Found Among 34910 Newborn Children: Results from a 13-year Incidence Study in �rhus, Denmark. Hum. Genet. 87, 81–83. 10.1007/bf01213097 2037286

[B17] Pierce K. Fitzgerald L. M. Seibel M. M. Zilberstein M. (1998). Preimplantation Genetic Diagnosis of Chromosome Balance in Embryos from a Patient with a Balanced Reciprocal Translocation. Mol. Hum. Reprod. 4, 167–172. 10.1093/molehr/4.2.167 9542975

[B18] Scriven P. N. Handyside A. H. Ogilvie C. M. (1998). Chromosome Translocations: Segregation Modes and Strategies for Preimplantation Genetic Diagnosis. Prenat. Diagn. 18, 1437–1449. 10.1002/(sici)1097-0223(199812)18:13<1437::aid-pd497>3.0.co;2-p 9949444

[B19] Stern C. Pertile M. Norris H. Hale L. Baker H. W. G. (1999). Chromosome Translocations in Couples with *In-Vitro* Fertilization Implantation Failure. Hum. Reprod. 14, 2097–2101. 10.1093/humrep/14.8.2097 10438432

[B20] Tan Y. Q. Tan K. Zhang S. P. Gong F. Cheng D. H. Xiong B. (2013). Single-nucleotide Polymorphism Microarray-Based Preimplantation Genetic Diagnosis Is Likely to Improve the Clinical Outcome for Translocation Carriers. Hum. Reprod. 28, 2581–2592. 10.1093/humrep/det271 23847111

[B21] Tan Y. Yin X. Zhang S. Jiang H. Tan K. Li J. (2014). Clinical Outcome of Preimplantation Genetic Diagnosis and Screening Using Next Generation Sequencing. GigaSci 3, 30. 10.1186/2047-217x-3-30 PMC4326468 25685330

[B22] Tease C. Hartshorne G. M. Hultén M. A. (2002). Patterns of Meiotic Recombination in Human Fetal Oocytes. Am. J. Hum. Genet. 70, 1469–1479. 10.1086/340734 11992253 PMC379134

[B23] Wang J. Li D. Xu Z. Diao Z. Zhou J. Lin F. (2019). Analysis of Meiotic Segregation Modes in Biopsied Blastocysts from Preimplantation Genetic Testing Cycles of Reciprocal Translocations. Mol. Cytogenet. 12, 11. 10.1186/s13039-019-0423-7 30858883 PMC6390622

[B24] Wang L. Shen J. Cram D. S. Ma M. Wang H. Zhang W. (2017). Preferential Selection and Transfer of Euploid Noncarrier Embryos in Preimplantation Genetic Diagnosis Cycles for Reciprocal Translocations. Fertil. Sterility 108, 620–627. 10.1016/j.fertnstert.2017.07.010 28863935

[B25] Warburton D. (1991). De Novo balanced Chromosome Rearrangements and Extra Marker Chromosomes Identified at Prenatal Diagnosis: Clinical Significance and Distribution of Breakpoints. Am. J. Hum. Genet. 49, 995–1013. 1928105 PMC1683246

[B26] Xie P. Hu L. Tan Y. Gong F. Zhang S. Xiong B. (2019). Retrospective Analysis of Meiotic Segregation Pattern and Interchromosomal Effects in Blastocysts from Inversion Preimplantation Genetic Testing Cycles. Fertil. Sterility 112, 336–342. 10.1016/j.fertnstert.2019.03.041 31103288

[B27] Xu J. Zhang Z. Niu W. Yang Q. Yao G. Shi S. (2017). Mapping Allele with Resolved Carrier Status of Robertsonian and Reciprocal Translocation in Human Preimplantation Embryos. Proc. Natl. Acad. Sci. U S A. 114, E8695–E8702. 10.1073/pnas.1715053114 28973897 PMC5642733

[B28] Ye Y. Qian Y. Xu C. Jin F. (2012). Meiotic Segregation Analysis of Embryos from Reciprocal Translocation Carriers in PGD Cycles. Reprod. BioMedicine Online 24, 83–90. 10.1016/j.rbmo.2011.08.012 22116068

[B29] Zhang C. Zhang C. Chen S. Yin X. Pan X. Lin G. (2013). A Single Cell Level Based Method for Copy Number Variation Analysis by Low Coverage Massively Parallel Sequencing. PLoS One 8, e54236. 10.1371/journal.pone.0054236 23372689 PMC3553135

[B30] Zhang L. Jiang W. Zhu Y. Chen H. Yan J. Chen Z.-J. (2019a). Effects of a Carrier's Sex and Age on the Segregation Patterns of the Trivalent of Robertsonian Translocations. J. Assist. Reprod. Genet. 36, 1963–1969. 10.1007/s10815-019-01534-6 31392661 PMC6730718

[B31] Zhang L. Wei D. Zhu Y. Jiang W. Xia M. Li J. (2019b). Interaction of Acrocentric Chromosome Involved in Translocation and Sex of the Carrier Influences the Proportion of Alternate Segregation in Autosomal Reciprocal Translocations. Hum. Reprod. 34, 380–387. 10.1093/humrep/dey367 30576528

[B32] Zhang S. Lei C. Wu J. Sun H. Zhou J. Zhu S. (2018). Analysis of Segregation Patterns of Quadrivalent Structures and the Effect on Genome Stability during Meiosis in Reciprocal Translocation Carriers. Hum. Reprod. 33, 757–767. 10.1093/humrep/dey036 29579270

[B33] Zhang S. Luo K. Cheng D. Tan Y. Lu C. He H. (2016). Number of Biopsied Trophectoderm Cells Is Likely to Affect the Implantation Potential of Blastocysts with Poor Trophectoderm Quality. Fertil. Sterility 105, 1222–1227. 10.1016/j.fertnstert.2016.01.011 26820770

[B34] Zhou S. Cheng D. Ouyang Q. Xie P. Lu C. Gong F. (2018). Prevalence and Authenticity of De-novo Segmental Aneuploidy (>16 Mb) in Human Blastocysts as Detected by Next-Generation Sequencing. Reprod. BioMedicine Online 37, 511–520. 10.1016/j.rbmo.2018.08.006 30228073

