# Supplementary figures and images for: Risk Factors Affecting Alternate Segregation in Blastocysts From Preimplantation Genetic Testing Cycles of Autosomal Reciprocal Translocations
Source: Front Genet. 2022 Jun 2;13:880208. doi: 10.3389/fgene.2022.880208 (PMC9201810; doi:10.3389/fgene.2022.880208)

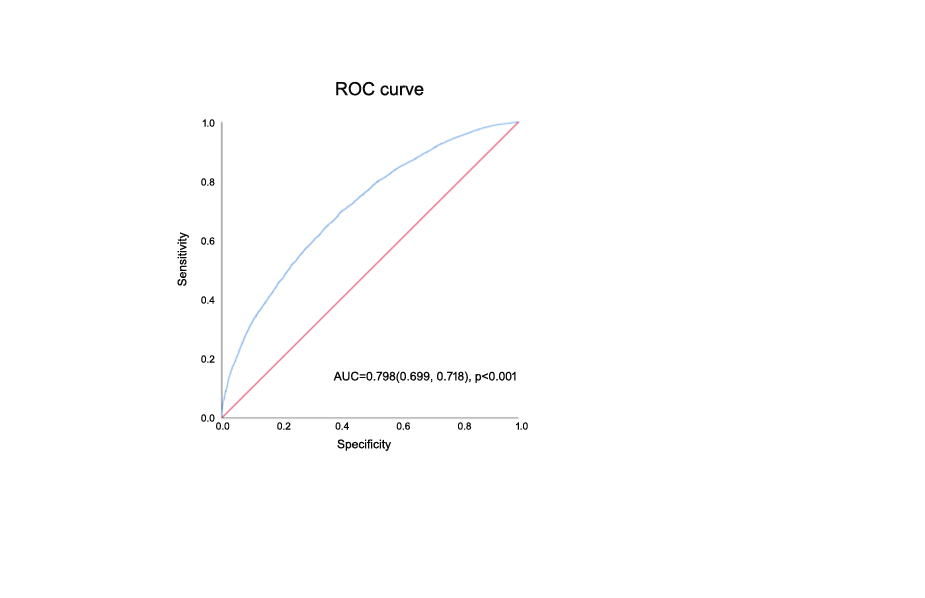

Supplement: Supplementary file 1 [file Image1.tif]
