# Supplementary material for: Risk Factors Affecting Alternate Segregation in Blastocysts From Preimplantation Genetic Testing Cycles of Autosomal Reciprocal Translocations
Source: Front Genet. 2022 Jun 2;13:880208. doi: 10.3389/fgene.2022.880208 (PMC9201810; doi:10.3389/fgene.2022.880208)
Supplement: Supplementary file 2 [file DataSheet1.PDF]

Supplementary Table 2 Alternate segregation proportion in male and female carriers according to the chromosome involved

| | With acr-ch | | | | | | | Without acr-ch | | | | | | |
|---|---|---|---|---|---|---|---|---|---|---|---|---|---|---|
| | Male | | | Female | | | | Male | | | Female | | | |
| | Total | Alt | % | Total | Alt | % | P-value | Total | Alt | % | Total | Alt | % | P-value |
| chr1 | 119 | 58 | 48.7% | 133 | 68 | 51.1% | >0.05 | 620 | 318 | 51.3% | 593 | 289 | 48.7% | >0.05 |
| chr2 | 140 | 93 | 66.4% | 127 | 58 | 45.7% | <0.01 | 577 | 295 | 51.1% | 571 | 259 | 45.4% | >0.05 |
| chr3 | 141 | 72 | 51.1% | 158 | 76 | 48.1% | >0.05 | 507 | 228 | 45.0% | 616 | 295 | 47.9% | >0.05 |
| chr4 | 126 | 56 | 44.4% | 108 | 31 | 28.7% | <0.05 | 482 | 232 | 48.1% | 714 | 338 | 47.3% | >0.05 |
| chr5 | 130 | 61 | 46.9% | 124 | 54 | 43.5% | >0.05 | 338 | 191 | 56.5% | 576 | 265 | 46.0% | <0.01 |
| chr6 | 98 | 58 | 59.2% | 99 | 27 | 27.3% | <0.05 | 362 | 176 | 48.6% | 540 | 239 | 44.3% | >0.05 |
| chr7 | 133 | 59 | 44.4% | 207 | 74 | 35.7% | >0.05 | 535 | 255 | 47.7% | 596 | 258 | 43.3% | >0.05 |
| chr8 | 81 | 38 | 46.9% | 197 | 72 | 36.5% | >0.05 | 355 | 179 | 50.4% | 531 | 211 | 39.7% | <0.01 |
| chr9 | 104 | 63 | 60.6% | 97 | 31 | 32.0% | <0.01 | 378 | 215 | 56.9% | 352 | 171 | 48.6% | <0.05 |
| chr10 | 89 | 48 | 53.9% | 74 | 21 | 28.4% | <0.01 | 405 | 192 | 47.4% | 596 | 275 | 46.1% | >0.05 |
| chr11 | 247 | 102 | 41.3% | 326 | 131 | 40.2% | >0.05 | 305 | 143 | 46.9% | 375 | 187 | 49.9% | >0.05 |
| chr12 | 69 | 35 | 50.7% | 93 | 35 | 37.6% | >0.05 | 279 | 156 | 55.9% | 450 | 193 | 42.9% | <0.01 |
| chr13 | 488 | 234 | 48.0% | 735 | 236 | 32.1% | <0.01 | \ | \ | \ | \ | \ | \ | \ |
| chr14 | 371 | 183 | 49.3% | 452 | 159 | 35.2% | <0.01 | \ | \ | \ | \ | \ | \ | \ |
| chr15 | 337 | 181 | 53.7% | 461 | 210 | 45.6% | <0.05 | \ | \ | \ | \ | \ | \ | \ |
| chr16 | 45 | 22 | 48.9% | 52 | 20 | 38.5% | >0.05 | 226 | 124 | 54.9% | 298 | 162 | 54.4% | >0.05 |
| chr17 | 77 | 40 | 51.9% | 70 | 27 | 38.6% | >0.05 | 258 | 138 | 53.5% | 236 | 128 | 54.2% | >0.05 |
| chr18 | 43 | 22 | 51.2% | 46 | 31 | 67.4% | >0.05 | 231 | 99 | 42.9% | 403 | 163 | 40.4% | >0.05 |
| chr19 | 17 | 8 | 47.1% | 27 | 6 | 22.2% | >0.05 | 156 | 79 | 50.6% | 119 | 66 | 55.5% | >0.05 |
| chr20 | 44 | 25 | 56.8% | 87 | 25 | 28.7% | <0.01 | 204 | 104 | 51.0% | 125 | 53 | 42.4% | >0.05 |
| chr21 | 169 | 82 | 48.5% | 184 | 66 | 35.9% | <0.05 | \ | \ | \ | \ | \ | \ | \ |
| chr22 | 416 | 193 | 46.4% | 407 | 173 | 42.5% | >0.05 | \ | \ | \ | \ | \ | \ | \ |

Supplementary Table 3 The proportion of adjacent-1 segregation under different values of TAR1 and carrier's sex group.

| Group | ≤0.2 | >0.2 | p-value |
|---|---|---|---|
| Overall | | | |
| Adjacent-1(%) | 1593(35.1%) | 1807(28.5%) | <0.01 |
| diagnosed embryos | 4533 | 6342 | |
| Female | | | |
| Adjacent-1(%) | 860(34.8%) | 974(27.7%) | <0.05 |
| diagnosed embryos | 2472 | 3513 | |
| Male | | | |
| Adjacent-1(%) | 733(35.6%) | 833(29.4%) | <0.01 |
| diagnosed embryos | 2061 | 2829 | |

$\chi^2$ was used to compare the differences between the frequencies of segregation products.

Supplementary Table 4 The proportion of adjacent-2 segregation in female and male carrier with reciprocal translocation.

| Group | Female | Male | p-value |
|---|---|---|---|
| Overall | | | |
| Adjacent-2(%) | 881(14.7%) | 467(9.6%) | $<0.01$ |
| diagnosed embryos | 5958 | 4890 | |

$\chi^2$ was used to compare the differences between the frequencies of segregation products.

Supplementary Table 5 The proportion of 3:1 segregation under different heterozygote's sex and chromosome type involved.

| Group | Female | Male | P-value |
|---|---|---|---|
| Overall | | | |
| 3:1 (%) | 429(7.2%) | 152(3.1%) | <0.01 |
| diagnosed embryos | 5985 | 4890 | |
| With acr-ch | | | |
| 3:1(%) | 199(9.3%) | 64(3.6%) | <0.01 |
| diagnosed embryos | 2129 | 1774 | |
| Without acr-ch | | | |
| 3:1 (%) | 230(6.0%) | 88(2.8%) | <0.01 |
| diagnosed embryos | 3856 | 3116 | |

$\chi^2$ was used to compare the differences between the frequencies of segregation products.
